# An Acupuncture Research Protocol Developed from Historical Writings by Mathematical Reflections: A Rational Individualized Acupoint Selection Method for Immediate Pain Relief

**DOI:** 10.1155/2013/256754

**Published:** 2013-01-29

**Authors:** Sven Schroeder, Gesa Meyer-Hamme, Jianwei Zhang, Susanne Epplée, Thomas Friedemann, Weiguo Hu

**Affiliations:** ^1^HanseMerkur Center for Traditional Chinese Medicine at the University Medical Center Hamburg-Eppendorf, Martinistrasse 52, House O55, 20246 Hamburg, Germany; ^2^Group TAMS, Department of Informatics, Faculty of Mathematics, Informatics and Natural Science, University of Hamburg, Vogt-Kölln-Strasse 30, 22527 Hamburg, Germany; ^3^World Federation of Acupuncture Societies, B-701, Dongjiu Building, Xizhaosi Street, Dongcheng Distict, Beijing 100061, China

## Abstract

While balancing yin and yang is one basic principle of Chinese medicine, balancing methods for combination of meridians and acupoints had been described throughout the history of Chinese medicine. We have identified six historical systems for combinations of acupuncture points in historical writings. All of them represent symmetrical combinations which are defined by the steps in the Chinese Clock. Taking the historical systems as a basis, we calculated the possible combinations that fit into these systems they revealed, leading to a total of 19 systems offering new balancing combinations. Merging the data of these 19 systems, there are 7 combinatorial options for every meridian. On the basis of this data, we calculated 4-meridian combinations with an ideal balance pattern, which is given when all meridians balance each other. We identified 5 of these patterns for every meridian, so we end up with 60 patterns for all the 12 meridians but we find multiple overlapping. Finally, 15 distinct patterns remain. By combining this theoretical concept with the *Image and Mirror Concept*, we developed an acupuncture research protocol. This protocol potentially solves some problems of acupuncture trials because it represents a rational reproducible procedure independent of examiner experience, but the resulting treatment is individualized.

## 1. Introduction

In Chinese Medicine, disease is understood as a loss of balance between the yin and yang energies [1, 2]. Balancing yin and yang is a basic concept of acupuncture treatment [1, 3], so combinations of meridians (or their corresponding organs) and acupoints for the balance of yin and yang had been described throughout the history of Chinese medicine [4]. Meridians are classified as yin or yang meridians. Lung (LU), spleen (SP), heart (HT), kidney (KI), pericardium (PC), and liver (LR) are defined as yin meridians, while large intestines (LI), stomach (ST), small intestines (SI), bladder (BL), triple energizer (TE), and gall-bladder (GB) are defined as yang meridians [5–10]. In our recent article [11] we have identified the known historical systems for combination of acupuncture points in historical writings and modern textbooks [3, 5–10, 12–27]. All of these represent symmetrical combinations, which were defined by the steps in the Chinese Clock (CC). In TCM theory, a continuous circulation of Qi through 12 meridians in a distinct order (LU, LI, ST, SP, HT, SI, BL, KI, PC, TE, GB, LR) is postulated, described as the Chinese medicine body clock, or Chinese clock (CC). 

### 1.1. Historical Systems

**Figure 1:**
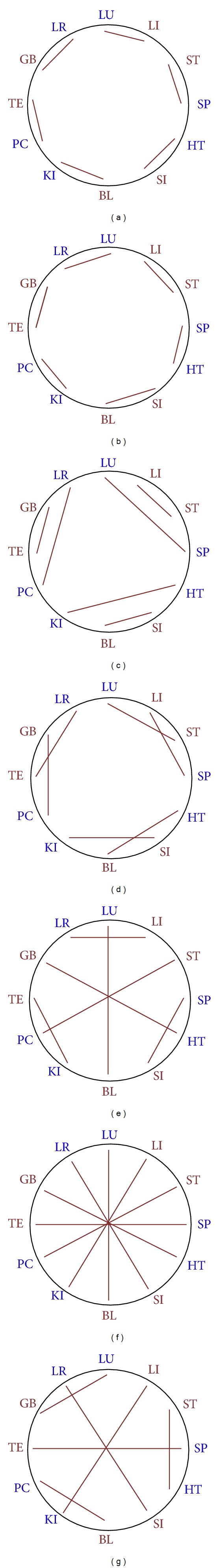
Graphical plotting of the historical systems. (a) Interior/exterior; (b) neighbouring channels; (c) 6-stage I; (d) 6-stage II; (e) 6-stage III; (f) opposite clock and one new system; (g) 6-stage IV. Blue: yin meridians, red: yang meridians.

The most common system is the *interior-exterior system*, a single-step system [17, 28–30]. It originates from Ling Shu (Ch. 2, Vol. 1) [23] and was described in detail in *The systematic classic of acupuncture and moxibustion* (Zhen Jiu Jia Yi Jing, Book 9) [17].  Figure 1(a) shows the plotting of the interior-exterior system on the CC. The* neighbouring channels system* is the second option of combining channels in a single-step system. It leads to arm-leg combinations of two yin or two yang channels [30–33].  Figure 1(b) shows the plotting of the neighbouring channels system on the CC. Three systems follow the theory of the 6 stages, originating from the Suwen (Chapter 6, (77 + 79)) and Lingshu (Chapter 5 (948 + 949) [10, 23] and have been described in detail in the Shanghanlun [10]. Since the description in *The Systematic Classic of Acupuncture and Moxibustion* [17] meridians are named according to the stages and the extremity where the main part of this meridian is running (Hand Tai Yang (SI), Foot Tai Yang (BL), Hand Yang Ming (LI), Foot Yang Ming (ST), Hand Shao Yang (TE), Foot Shao Yang (GB), Hand Tai Yin (LU), Foot Tai Yin (SP), Hand Jue Yin (PC), Foot Jue Yin (LR), Hand Shao Yin (HT), and Foot Shao Yin (KI)). Combining the meridians of one stage is very popular and is called “anatomical system” in some schools [34]. This system represents a 1-step–3-step alternating system. We call it the *6-stage system I*.  Figure 1(c) shows the plotting of the 6-stage system I on the CC. The next system we call *6-stage system II*. It combines stages Tai Yang and Shao Yin, stages Yang Ming and Tai Yin as well as stages Shao Yang and Jue Yin. It is widely used in modern schools [18, 29, 31, 32].  Figure 1(d) shows the plotting of the 6-*stage system II* on the CC.

*6-stage system III* originates from the Ming-Dynasty [18]. This system combines Tai Yang with Tai Yin, Jue Yin with Yang Ming and Shao Yang with Shao Yin.  Figure 1(e) shows the plotting of the 6-stage system III on the CC.

Cross needling originates from Chapter 63 of the Suwen and is called opposite clock needling in modern school [30] and also used in Japanese acupuncture [18].  Figure 1(f) shows the plotting of the *opposite clock system* on the CC. 

### 1.2. Intrinsic Rules of the Historical Systems

All historically described systems have in common the fact that they build a symmetrical combination in the CC with a rotation symmetry of 30°, 60° or 120°. Every meridian pairs with only one other and no meridian is left over, so there are always 6 pairs of meridians. A maximum of two alternating steps are used, leading to yin-yang/yang-yin or to yin-yin/yang-yang combinations. They can be described as intrinsic rules of the historical systems, summarized in Table 1. A graphical plotting of all historical systems is shown in Figures 1(a)–1(f).

### 1.3. Graph Traversal Search for Identification of Further Systems

Our question was to find out whether there are more systems than historically described and whether any meridians can be excluded as potentially balancing meridians, so we calculated all symmetrical combinatorial possibilities. We did a graph traversal search for further systems, described in detail in our recent paper, available online [11]. 19 of the symmetrical patterns followed the intrinsic rules of the historical systems (Table 2).

### 1.4. Additional Combinations from Graph Traversal Search

One 2-step–6-step alternating system we want to emphasize, since it combines stages Tai Yang and Jue Yin, Yang Ming and Shao Yin, as well as Shao Yang and Tai Yin. So we named it *6-stage system IV* (Figure 1(g)).

There are three more 1-step–3-step alternating systems that lead to additional point combinations not covered by those historically described.

Step 5 provides an additional combinatorial possibility, but has no extensive tradition in Chinese medicine except for concepts connected to the extraordinary vessels. All pairs of Yin extraordinary vessels can be opened by using a 5-step combination which includes the very commonly used technique to combine the master points of the paired extraordinary vessels [35]. The master point of the primarily treated extraordinary vessel is needled at first, then the master point of the paired extraordinary vessel, called the coupled point, is needled secondly. Chong Mai (SP-4, Gongsun) and Yin Wei Mai (PC-6, Neiguan), Ren Mai (LU-7, Lique), and Yin Qiao Mai (KI-6, Zhaohai) represent this 5-step combination [24, 35, 36].

With step 5 distant parts of the body are connected. A combination of step 5 with another step in the Chinese Clock is mathematically not possible, if the intrinsic rules of the historical system are to be observed. As described above this is, however, possible for the 1–3-step and for 2–6-step combinations of two steps for an balance in accord with the intrinsic rules (Table 2). This might be an explanation why the points of the extraordinary vessels are usually used alone in a two-point combination [35, 36]. So if you combine a step 5 combination with other meridians, there is a high risk of applying an unbalanced treatment. 

The possibilities for finding a balanced treatment strategy can be described by the steps that have to be taken in the Chinese clock to combine acupuncture points. Merging the data of all systems, the steps in the Chinese clock showing a possibility for balancing are the following. Steps 1, 2, and 3 are possible so is step 6. Step 4 is not a combinatorial possibility. Step 5 is a combinatorial possibility, but has no tradition in TCM, except in the theory of the extraordinary vessels [35, 36], might lead to an imbalance in multipoint combinations. A summary is given in Table 3.

**Figure 2:**
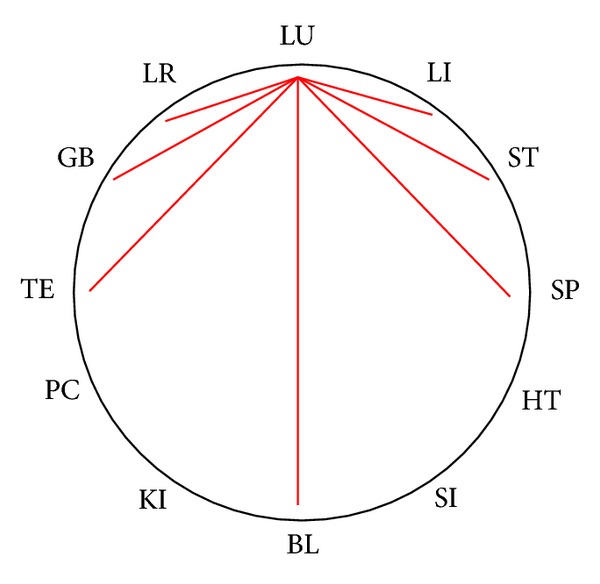
Merging of the combinatorial possibilities (this can be done with every meridian in a similar way).

In Table 4 we list all of the 19 calculated systems, that allow a multimeridian combination. This includes the historical systems, marked in yellow. Systems that allow new combinations are marked in blue, while calculated systems that do not offer new combinations, that are already known by the historical systems, are not marked. The merged data plotted on the CC are shown as an example for the meridians of the lung (see Figure 2). This can be applied for all meridians in a similar way. It shows clearly, that every meridian can be balanced by 7 other meridians. Plotted on the CC these seven options are always step 1, 2, 3 clockwise and counterclockwise as well as step 6. For daily practice this is helpful to quickly identify the treatment options for balancing the affected meridian. It can be used as a tool for quick memorization.

For a TCM practitioner these 7 options might be mainly understood as energetic relationships. We describe this for the example of the LU-Meridian, plotted on the CC in Figure 2. Step 1 clockwise is the internal (LU)-external (LI) relationship, Step 2 clockwise is the Hand-Tai Yin (LU)-Foot-Yang Ming (ST) relationship, described by *6-stage system II*. Step 3 clockwise is the Hand-Tai Yin (LU)-Foot-Tai Yin (SP) from *6-Stage system I*. Step 1 counterclockwise is the *Neighbouring channels* relationship LU-LR, Step 2 counterclockwise is the Hand-Tai Yin (LU)-Foot-Shao Yang (GB) described in the new system *6-stage IV*. Step 3 counter clockwise describes a previously not described combination deriving from mathematical calculations with no obvious theoretical systematic background. Step 6 describes the opposite-clock relationship of LU and BL.

### 1.5. Combination of More than Two Meridians

Merging of the combinatorial possibilities leads to 7 options for a balanced treatment for every meridian. It might lead to the idea that “everything goes” in acupuncture. This is actually not true. If only two meridians are combined there are these 7 options. Nevertheless, it is very common to combine more than 2 meridians in an acupuncture treatment. For this reason keeping a treatment balanced in accordance with the intrinsic rules of the historical systems becomes complicated. So some acupuncture schools start to describe patterns, usually combining two historical systems treating all four extremities [30–32, 37] to avoid unbalanced combinations.

**Figure 3:**
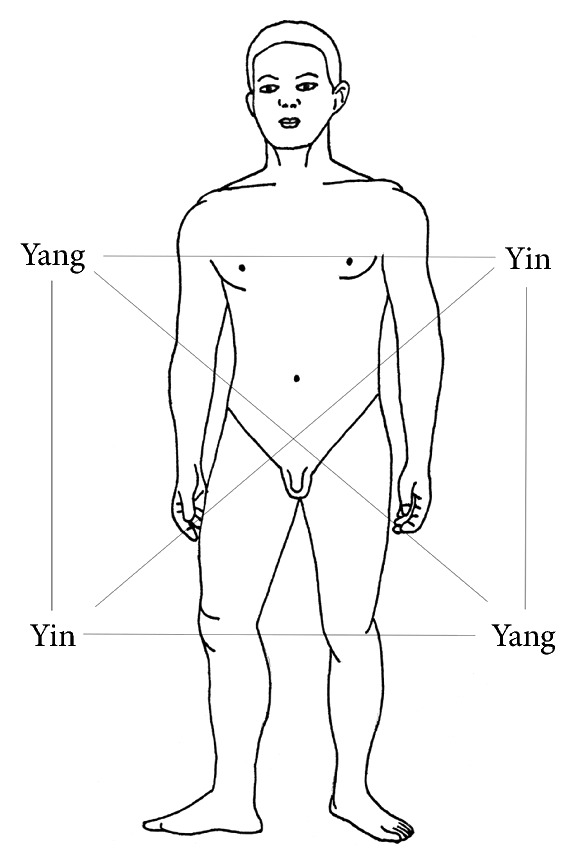
Balanced treatment in projection to the body.

So a complete balanced treatment of a meridian combines a left-right yin-yang balance and an up-down yin-yang balance (Figure 3). In this context the goal is to identify a pattern which will lead to a balance in which every meridian is balanced by all other meridians employed, in accordance with the above-described intrinsic rules of the historical systems. This is what we call an ideal balance.

### 1.6. Using Somatotopic Knowledge for the Search of the Right Points on a Balancing Meridian

**Figure 4:**
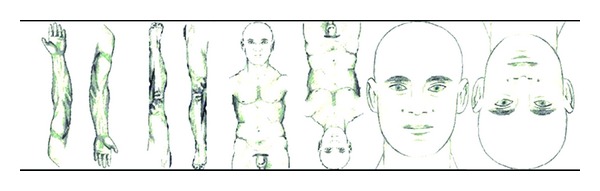
. Somatotopic image and mirror of different body areas.

Somatotopy is the mapping of touch, vibration, and heat signals coming from different parts of the body to distinct and specific locations in the brain's primary somatosensory cortex (SI). This somatotopic organization is sometimes described as the homunculus of the brain [38]. The somatotopic knowledge is old and is already described in detail in Su Wen (e.g., Chapters 67, 68, and 71) describing the relationship between up and down, left and right, front and back [23]. The most likely form of acupoint specificity lies in the somatotopic response [39]. Some modern schools devote great attention to these somatotopies and use them for point selection, calling this the *Image and Mirror Concept* [30–33]. This *Image and Mirror Concept* describes somatotopic projections of different body areas. It is very useful for the identification of possible areas for treatment, especially when combined with the knowledge of the balancing meridians. Image means in this context, that you can project a part of the body (e.g., the arm) on another part of the body (e.g., the leg) to identify a somatotopic connection. Mirror means in this context, that you also can project an upside-down picture of one body part to another body parts, to find somatotopic connections. So for example, effective points for treatment of the knee region can be found in the region of the elbow, chest, eyes, or nose. In Figure 4 you find a graphical plotting of somatotopically connected body areas.

## 2. Material and Methods

As a basis for further analysis we took the above described 2-meridian combinations, that follow the intrinsic rules of the historical systems. While the merging of the combinatorial possibilities leads to 7 options for every meridian for a balanced treatment, we were interested in combining more than 2 meridians in a balanced acupuncture procedure. We decided to calculate the mathematical options for combining 4 meridians to gain an ideal balance, because the human body has four extremities. We defined as an ideal balance a combination of meridians, in which every meridian is balanced by all other meridians employed, in accord with the above-described intrinsic rules of the historical systems.

First we calculated all the possible 4-meridian combinations of the 12 meridians in the CC. Second we reduced the number to the above described 7 options. We listed all possibilities. Third we identified and described the patterns that followed our definition of an ideal balance.

Finally we combined the resulting information with the knowledge of the above described *Image and Mirror Concept* for the development of an research protocol for acupuncture. 

## 3. Results

### 3.1. Combinatorial Possibilities

The 12 meridians in the circle were labelled 1, 2, 3,…, 12. There are 6 possible steps in the Chinese clock: 1, 2, 3, 4, 5, and 6. (e.g., step 1 is a connection of two neighbouring meridians, step 2 skips one, combining the first meridian with the third one, etc.). To connect 4 meridian, all solutions can be classified into 3 groups. Every meridian point pairs with other 3 and no meridian is left over. *C*_*n*_^*k*^ is the number of combinations of *n* things taken *k* at a time *n* : !/((*n* − *k*)!*k*!. The possible combinatorial number is *C*_12_^4^ *C*_8_^4^ *C*_4_^4^/(3!) = 5775.

Then the step of each group was calculated. There are 6 possible steps in the Chinese Clock: 1, 2, 3, 4, 5, 6, as is shown in Figure 1. The number of possible combinations was counted. 4-point combination where every point is a combinatory possibility with every other, following the rule of 2-point combinations, where every point only can be combined with 7 others, as is shown in Figure 2. The combinations with steps 4 and 5 were eliminated, because they did not correspond to the intrinsic rules of the historical system. So the possible steps were only 1, 2, 3, and 6. There are only 4 kinds of steps, though here are 7 options of combination (as shown in Figure 2) for every meridian.

### 3.2. Development of 4-Meridian Patterns

An ideal balance pattern for meridian-balancing is given, when all meridians balance each other. So an ideally balanced 4-meridian-pattern is only present, if every meridian is a possible combination (according to one of the 7 options) of every other meridian of this 4-meridian pattern. So we manually evaluated the 4-point combinations according to Table 4.

**Figure 5:**
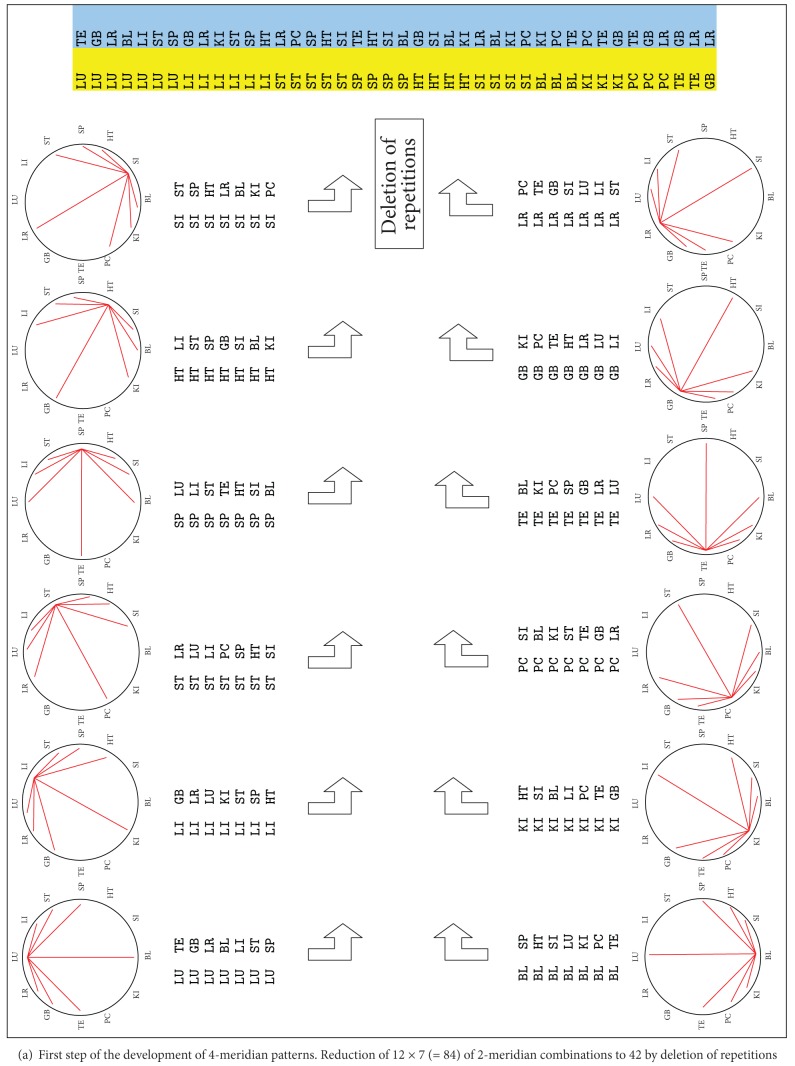

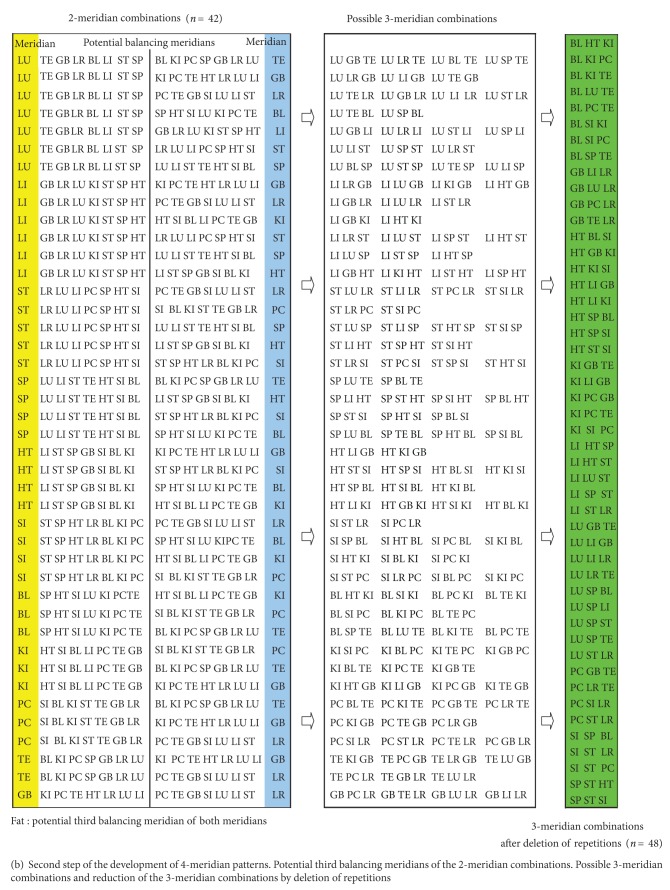

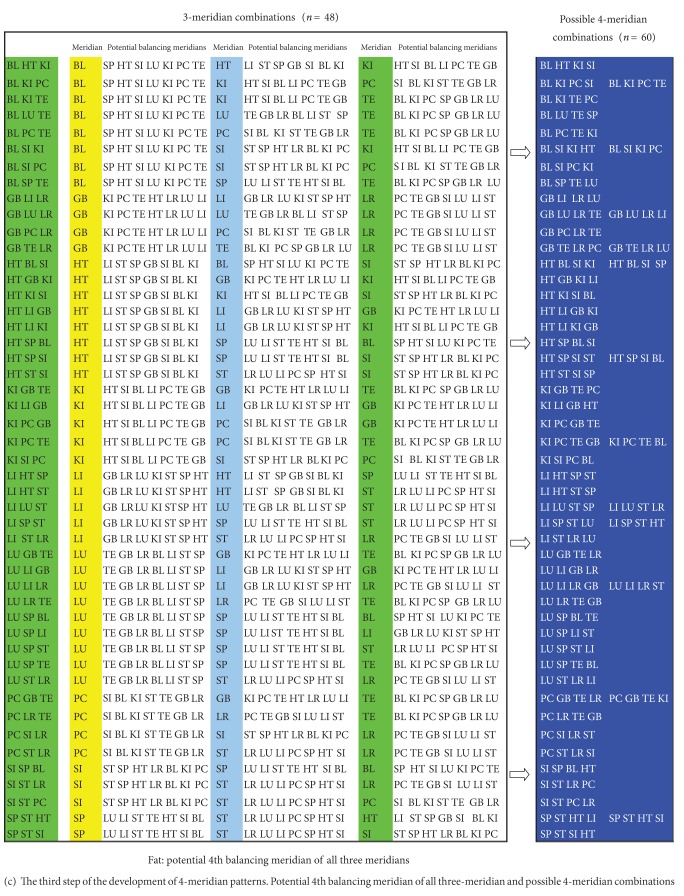

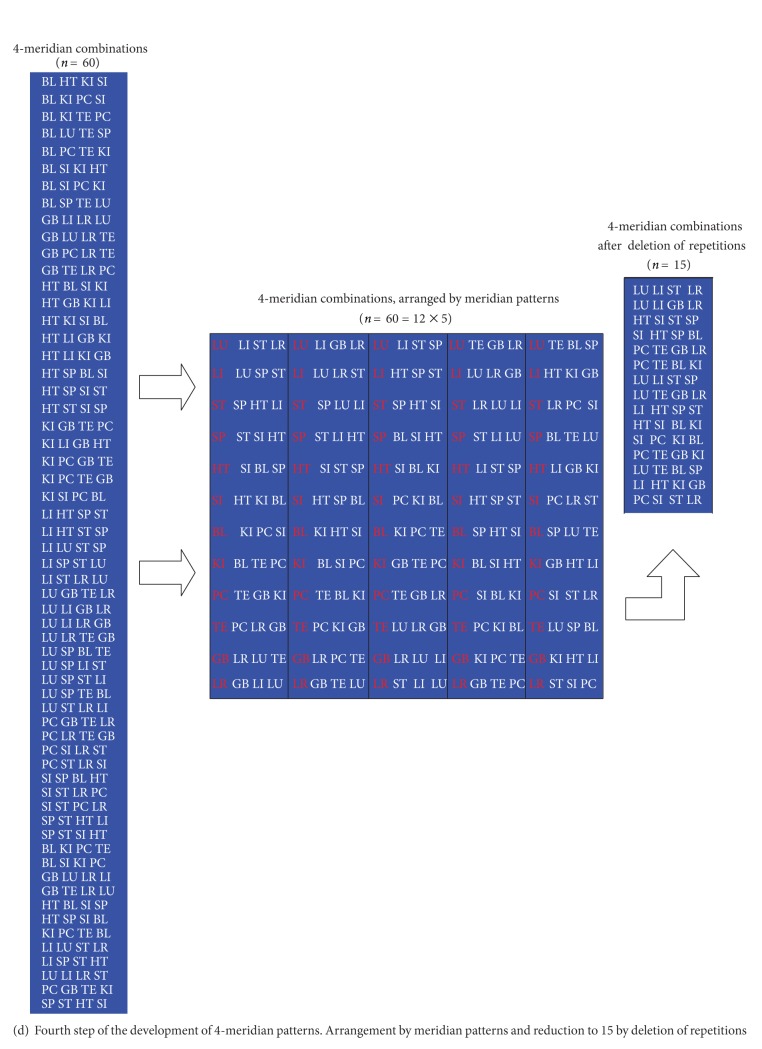


We listed all possibilities by plotting the combinations on the CC. While there are 12 meridians, there are 12 × 7 = 84 options for 2-meridian combinations. These were manually evaluated and the repetitions were deleted, resulting a reduction to 42 options of 2-meridian combinations. This first step of the development of 4-meridian patterns is shown in Figure 5(a).

These 42 pairs of meridians were evaluated for their common potential balancing meridians. We found 24 times 4 shared balancing meridians for the pairs of meridians, 12 times 3 shared balancing meridians and 6 times 2 shared balancing meridians, leading to 144 options for 3-meridian combinations. We deleted the repetitions and found 48 unique options for 3-meridian combinations.

This second step of the development of 4-meridian patterns is shown in Figure 5(b).

The 48 3-meridian combinations were evaluated for their common potential balancing meridians. We found 12 times 2 shared balancing meridians of all 3 meridians and 48 times one shared balancing meridian of all 3 meridians, leading to 60 possible 4-meridian combinations. This third step of the development of 4-meridian patterns is shown in Figure 5(c). 

These 60 possible 4-meridian combinations were manually new arranged by the common patterns on the CC and their connection to the 12 meridians. This is shown in Figure 5(d).

For every meridian five ideally balanced 4-meridian-patterns could be identified. They all had a similar pattern on the CC. This is shown in Table 5, *x* is the meridian which is to be balanced; the numbers are the steps in CC, + means clockwise, and − means counterclockwise.

**Figure 6:**
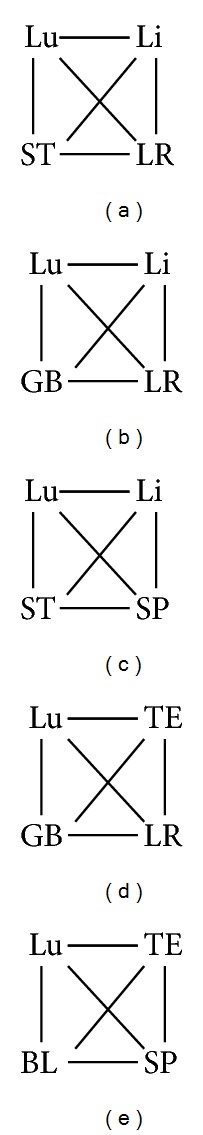
Possible patterns for balancing the LU-meridian.

**Figure 7:**
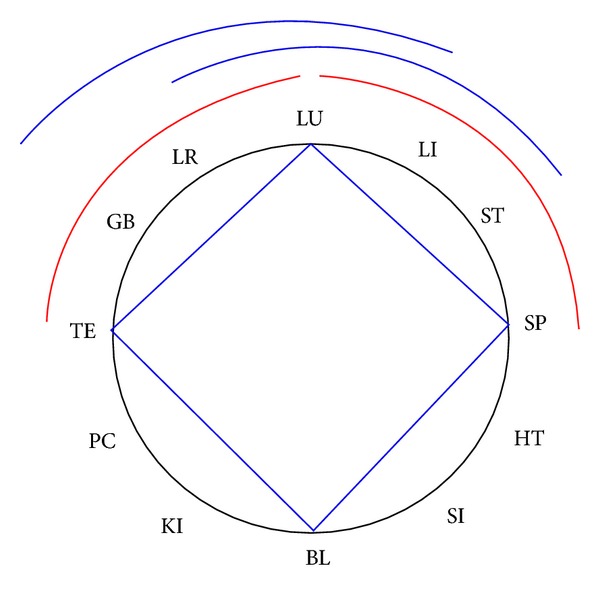
Graphical plotting of the 5 pattern for balancing the lung meridian on the Chinese Clock.

There are always only five ideal balanced options by applying treatment to the three not effected extremities and optionally to the effected meridian itself. So ideal balanced patterns can be plotted similar to Figure 3 with a left-right yin-yang balance and an up-down yin-yang balance. An example for the 5 patterns of the LU-meridian is shown in Figure 6. The pattern can also be plotted on the CC, as shown in Figure 7.

**Figure 8:**
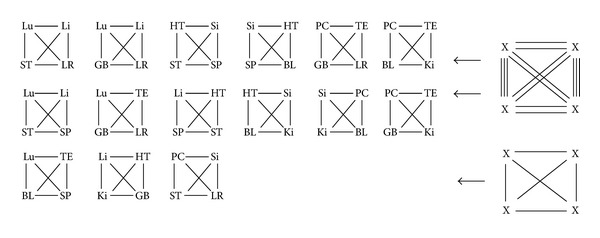
15 ideally balanced patterns.

So every meridian can be balanced by 5 different patterns, but we find multiple overlapping. If we delete all repetitions, 15 patterns remain. This is shown as the fourth step of the development of 4-meridian patterns in Figure 5(d). These 15 patterns are plotted a left-right yin-yang-balance and an up-down yin-yang balance in Figure 8. Interestingly all patterns plotted in row 1 and 2 have a balance in two or three of the calculated systems, while all patterns in the third row have only a single balance in one of the above described systems (Table 4).

**Figure 9:**
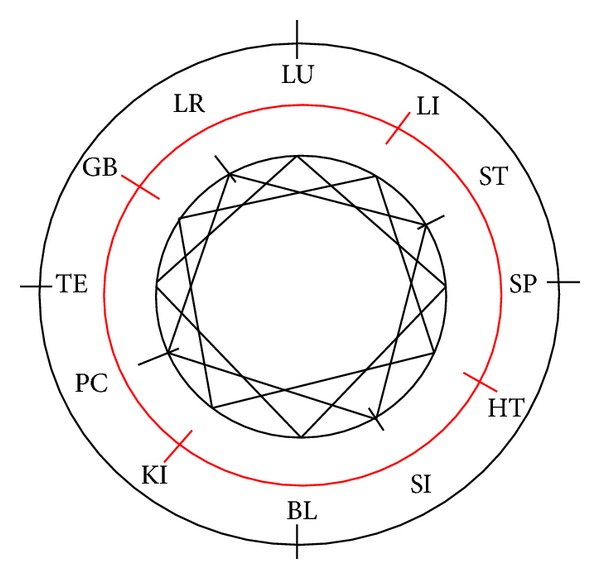
Graphical plotting on the Chinese Clock of 15 ideally balanced patterns.

A graphical plotting, shown in Figure 9, shows that all patterns together build a symmetrical picture on the CC.

### 3.3. Research Protocol for Pain Relief in Localised Pain

Extremities include not only hand and arm or leg and foot, but as well parts of meridians on trunk, neck, or face of the meridian connected to and named by the extremity (hand or foot). The knowledge of finding the right balancing meridians is even more useful, if it is combined with the somatotopic knowledge of the *Image and Mirror Concept* for finding the right points and the right area on the chosen meridian. In combination, these two methods are extremely useful in the treatment of localized pain and can often produce immediate effects. For best results, first the affected meridian in the region of pain must be identified, then it is detected and treated using a corresponding meridian (according to Table 4) in a corresponding area on an unaffected extremity (according to Figure 4) and then the result is checked. In case a change of symptoms but not yet complete pain reduction is found, a corresponding meridian on another extremity should be chosen and treated in the same way until a satisfactory result in the painful area is achieved.  Table 6 summarizes the practical approach.

**Figure 10:**
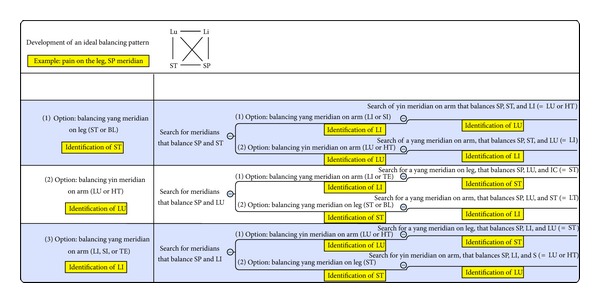
Development of an ideal balance pattern.

Even though possibilities of choice are listed in the protocol, different approaches lead to similar patterns and success rates. For the example of one pattern see the description in Figure 10.

### 3.4. Case Example

A 60-year-old carpenter worked overhead for a full day and developed severe shoulder pain. Pain was prominent on the dorsal head of his shoulder. Pain was increased by lateral lifting of the shoulder and by inward rotation of the arm. Pain could be exaggerated by pressure over the lateral scapula; muscles around this area were tight. The active range of movement of the right shoulder was reduced. The shoulder could only be laterally lifted to 50° due to severe pain.

The patient was asked to estimate the pain on a visual analog scale (VAS) from 1 to 10. He described his pain as 9/10 on VAS.

*The Right SI-Meridian Was Identified as the Affected Meridian*

The area of maximum pain was at point SI-10 (Naoshu) and 2 cun above and below this point. It was decided to start on the legs first and start with a Yang meridian. According to Table 4 the BL- and the ST-meridian are balancing Yang meridians of the SI-meridian. Turning Figure 2 to the IT-meridian, the ST-meridian is the third step from IT if you go clockwise and the BL-meridian is the first step from IT, if you go counterclockwise. These are the only possible yang meridians of the lower extremities. Applying concept on the lower extremities the region around the ankle, the hip region and the region above and below the knee are potentially somatotopic regions of treatment. The BL- and ST-meridian was palpated on both legs with a special focus on the somatotopic regions described above for Ashi points. 

There was no region of pain on the ST-meridian. There were painful Ashi points on the BL-meridian, very mild above and below the knee but severe in the area of the ankle. The pain was much more prominent on the left side. 

*So It Was Decided to Treat the Left BL-Meridian*

The maximum area of Ashi was at left Bl-60 (Kunlun). A sterile 0.2 × 0.22 mm acupuncture needle was perpendicular inserted 0.5 cun at this point and short stimulated by reducing method. 2 other needles approximately 0.25 and 0.5 cun proximal and 2 other needles approximately 0.25 and 0.5 cun distal of Bl-60 on the BL-meridian in the maximum area of Ashi were inserted and stimulated in a similar manner. The patient was asked to move his arm. He was able to lift it laterally up to an angle of 90°. Painful areas were palpated again. The patient estimated his pain as 5/10 on VAS.

Then we looked at the right leg for a yin meridian that balances the SI- and the BL-meridian. According to Table 4 the KI- and the SP-meridians were candidates. The KI-meridian is the first step clockwise of the BL-meridian as well as the second step clockwise of the SI-meridian. The SP-meridian is the third step counterclockwise of the the BL-meridian as well as the second step of the SI-meridian. Applying *Image and Mirror concept* on the lower extremities we again palpated the KI- and SP-meridian for Ashi point in the region around the ankle, the hip region and the region above and below the knee. There was no region of pain on the KI meridian. There were painful Ashi points on the SP-meridian, very mild above and below the knee but severe in the area of the ankle. 

*So It Was Decided to Treat the Right SP-Meridian*

The maximum area of Ashi was at left SP-5 (Shangqiu). A sterile 0.2 × 0.22 mm acupuncture needle was perpendicular inserted 0.2 cun at this point and suppletively stimulated. 2 other needles approximately 0.25 and 0.5 cun proximal and 2 other needles approximately 0.25 and 0.5 cun distal of SP-5 on the spleen meridian in the maximum area of Ashi were inserted and stimulated in a similar manner. The patient was asked to move his arm. He was able to lift it laterally up to an angle of 110°. Painful areas were palpated again. The patient estimated his pain as 3/10 on VAS.

Then we looked for a yin meridian on the left arm that balances SI, BL and SP. According to Table 4 this is only the HT-meridian.The HT-meridian is the first step counterclockwise of the SI-meridian as well as the second step counterclockwise of the BL-meridian as well as the first step clockwise of the SP-meridian.

*So It Was Decided to Treat the Left HT-Meridian*

We palpated the HT-meridian of the left arm according to Image and Mirror, searching for Ashi points in the area of the shoulder, the wrist and above and below the elbow. The HT-meridian on the shoulder had some mild Ashi points, but the major region of Ashi was around HT-3 (Shaohai). A sterile 0.2 × 0.22 mm acupuncture needle was obliquely inserted 0.2 cun at this point and suppletively stimulated. 2 other needles approximately 0.25 and 0.5 cun proximal and 2 other needles approximately 0.25 and 0.5 cun on the HT-meridian were inserted and similarly stimulated.

The patient was asked to move his arm. He was able to lift it laterally up to an angle of 180°. Painful areas were palpated again. The patient estimated his pain as 1/10 on VAS.

Finally, the affected meridian itself was palpated according to image and mirror for Ashi points in the area of the wrist and above and below the elbow. 

*So It Was Decided to Treat the Affected SI-Meridian Itself*

The major region of Ashi was around SI-5 (Yanggu). A sterile 0.2 × 0.22 mm acupuncture needle was perpendicularly inserted 0.3 cun at this point and stimulated by reducing method. 2 other needles were inserted approximately 0.25 and 0.5 cun proximally; one needle was inserted into SI-4 (Wangu) and another needle approximately 0.25 cun distal on the SI-meridian. They were then stimulated as described above. 

The patient was asked to move his arm. He was still able to lift it laterally up to an angle of 180°. Painful areas were palpated again. The patient estimated his pain as less than 1/10 on VAS. 

*So in This Case the Pattern SI-HT-BL-SP Was Used*

The patient was given an appointment for the following day. At this time he reported a relapse in the previous night. He estimated his pain as 4/10 on VAS. A similar treatment was applied. After treatment he again estimated his pain as less than 1/10 on VAS. The next day he had his third appointment. His pain had not returned and was reported as less than 1/10 on VAS. He again got a similar treatment on all non-effected extremities. After this no pain was reported any more. His shoulder showed full active and passive movement in all directions. So no extra treatment on the effected meridian was applied.

The patient stayed pain-free and returned to work. 

## 4. Discussion

This paper is based on an analysis of the historically described balancing acupuncture systems found in historical writings and which have been recurrently described in modern textbooks. It uses a mathematical approach to calculate the theoretical options based on the historical systems as a first step. All these historical systems describe combinations of two meridians. They had been determined on the basis of empirical knowledge by generations of experts in Chinese Medicine. But while all the historical systems show a certain symmetry when plotted on the CC and while the order in the CC most likely put a distinct order due to anatomical reasons [11], the CC can be seen as a mathematically organized symmetric description of the three surfaces of the body (back, front, and medial/lateral) [11]. So a search, whether the historical systems offer a complete description of combinatorial possibilities, is only rational. The historically established systems for combining meridians cover many, but not all, of our calculated combinations of meridians. So our mathematical approach offers theoretically calculated supplementary information to the wisdom of generations of physicians, leading to 7 balancing options for every meridian.

Balancing yin and yang is a basic concept in Chinese medical theory and a basis for acupuncture treatment [1–3]. There is a risk of losing this balance, if the acupuncture plan involves more than two meridians. So as a second step we calculated the option for a balanced acupuncture treatment scheme with more than 2 meridians. Balancing up and down, left and right, and front and back has a long tradition in TCM based on the Suwen [23], and the human body has four extremities, we decided to calculate the mathematical options to gain an ideal balance for 4 meridians. An ideal balance means that every chosen meridian balances all other meridians.

All these approaches, determined by way of a mathematical search for combinatorial possibilities is based on theoretical considerations from historical writings and supported by empirical knowledge.

Our observations do not have to be considered as being the only concept in acupuncture. There are more possibilities like balancing yin and yang meridians on one extremity, applying bilateral treatment of the same meridian, there are microystems like scalp and ear acupuncture, as well as empirically determined point combinations, that do not follow the concept of balancing [29, 40, 41].

But the concept of of a balanced treatment has a long tradition in TCM and in the historical writings [23], so we consider it to be a rational basis for logical and reproducible acupuncture strategies.

The authors' experience of achievement of immediate effects in pain management by application of this protocol leads to the question of the mechanisms involved. Acupoints on very distant parts of the body, in some cases even with a completely different segmental innervation in the spinal column, can induce a therapeutic effect on local pain. This contradicts the theory that the main effect of acupuncture treatment is due to peripheral or segmental effects [42–44], so our experience supports the idea of other authors that higher-level central pathways play a central role in pain-releasing effects of acupuncture [45, 46]. But is has to considered, that this protocol is only based on theoretical reflections and has to be approved in clinical studies. 

Interestingly, there are 5 ideally balanced acupuncture patterns for every meridian. Four of them represent a balance that is found in 2 or 3 of the above calculated systems for every meridian pair (Table 2). These patterns are four neighbours in a row in the Chinese Clock.

In one pattern every balance derives from only one system in Table 2. It combines every third step in the CC. Further research has to be done to determine whether the different patterns represent typical clinical conditions and whether the one pattern with the single balance between 2 meridians represents an exceptional treatment option. 

Some schools describe rules whereby it is necessary to combine points by one of the above described systems on the contralateral or ipsilateral side, but no explanations are given [30–32]. Mathematically, at least for the calculated 4-point ideally balanced pattern, it could be either ipsilateral or contralateral retainment of the balance. According to our experience in the treatment of localised pain you have to palpate bilaterally for Ashi. The more sensitive area leads to the decision for contralateral or ipsilateral treatment.

Due to multiple overlapping of the 5 patterns for each meridian there only exist a total of 15 distinct patterns for all meridians. Pattern diagnosis has a long tradition in TCM. Syndromes or patterns are described as Zheng diagnosis [47]. TCM Zheng describes major patterns of vegetative disharmonies which are identified by using a comprehensive analysis of clinical information from four main diagnostic TCM methods: observation, listening, questioning, and pulse analysis [48].

Modern research on Zheng diagnosis is devoted to the search for a correlation between Zheng patterns and system biology parameters [48–50].

Further investigation of the correlation of the described ideally balanced acupuncture patterns and the Zheng patterns might lead to an improvement of clinical treatment. The 5 patterns for every meridian described above and the reduction to 15 patterns for all meridians due to overlapping might explain the overlapping of Zheng patterns, resulting in similar patterns to different diseases as well as different patterns for similar diseases [51].

Even though pain has been the focus of most clinical research on acupuncture [52], there are no controlled studies with hard data on the effect of balanced 4-point or meridian combinations in pain research so far. This is a necessity for future trials. By transferring this theoretical consideration into research we have been describing an empirical systematic approach for the achievement of immediate effects in the treatment of localised pain. This can be used as a research protocol for future trials as well as a treatment protocol. While it is a protocol for immediate effects, studies can be cost effective because it can reduce the number of treatments to one.

Acupuncture treatment based on TCM usually requires an individual diagnosis which leads to an individual treatment strategy [29]. This approach is in some aspects contradictory to controlled clinical trials in which treatment procedures are usually uniform. Trials with individual treatments are often considered to be less scientific. This is a severe problem for the design of studies on TCM treatments and an obstacle for publication in highly ranked journals. Otherwise TCM experts criticise that controlled trials do not reflect the practice of acupuncture. In controlled trials often no diagnostic framework is applied resulting in a lack of individualisation to address specific TCM imbalances and symptoms [53].

Our protocol potentially solves this problem because it offers a rational, reproducible procedure independent of examiner experience, but the resulting treatment procedure is an individual. This has many advantages. The necessary acupuncture pattern is developed during examination. The pattern of treatment is not fixed before treatment, so probands of the treatment group are not at risk, to get an ineffective treatment for their condition. While the treatment is developed in a rational examination process, the probands will have a good chance to get a corresponding treatment to their body reaction. This will increase the efficacy of the treatment procedures. Anyhow limitation of this protocol are, that it is only based on theoretical consideration and personal experience of the authors. It has to be further approved in clinical trials.

In addition to the test of the efficacy of acupuncture in pain management, information on patterns can be gained in studies following our research protocol. 

This might increase the knowledge as to which acupuncture patterns are most sufficient under which clinical conditions and lead to improved treatment strategies as well as to results that help increase the knowledge on the mechanisms of acupuncture. 

## 5. Conclusion

With this systematic description of a mathematical model for the calculation of combinatorial possibilities of meridians as well as the calculation of ideally balanced patterns we describe a theoretical model applicable for acupuncture research and treatment. By sharing our protocol for immediate effects in localized pain treatment we offer researchers a protocol for controlled acupuncture trials with an individualized approach based on historical knowledge. Further research has to approve this theoretical approach and might have influence on understanding mechanism of pain relief in acupuncture. By this approach, theoretical and empirical knowledge is organized into a systematic approach.

## Figures and Tables

**Table 1:** Intrinsic rules of the historical systems.

Every meridian pairs with only other one
Rotation symmetry of 30°, 60°, or 120°
6 pairs of meridian
Maximum of 2 alternating steps
6 yin/yang or 3 yin/yin and 3 yang/yang combinations

**Table 2:** Combinations that follow the intrinsic rules of the historical systems, listed according to steps in the Chinese Clock.

1-step systems	= 2
1-step–3-step alternating systems	= 4
2-step systems	= 4
2-step–6-step alternating systems	= 4
3-step systems	= 2
5-step systems	= 2
6-step systems	= 1

Total number	19

**Table 3:** Possible steps in the Chinese clock for balancing a meridian.

1, 2 and 3 are possible	
4 is not possible	
5 is possible, but has no tradition in TCM, except in the theory of the extraordinary vessels and might be unbalanced in a multiple point concept	
6 is possible	

**Table 4:** Systems for combinations of meridians for multimeridian combinations.

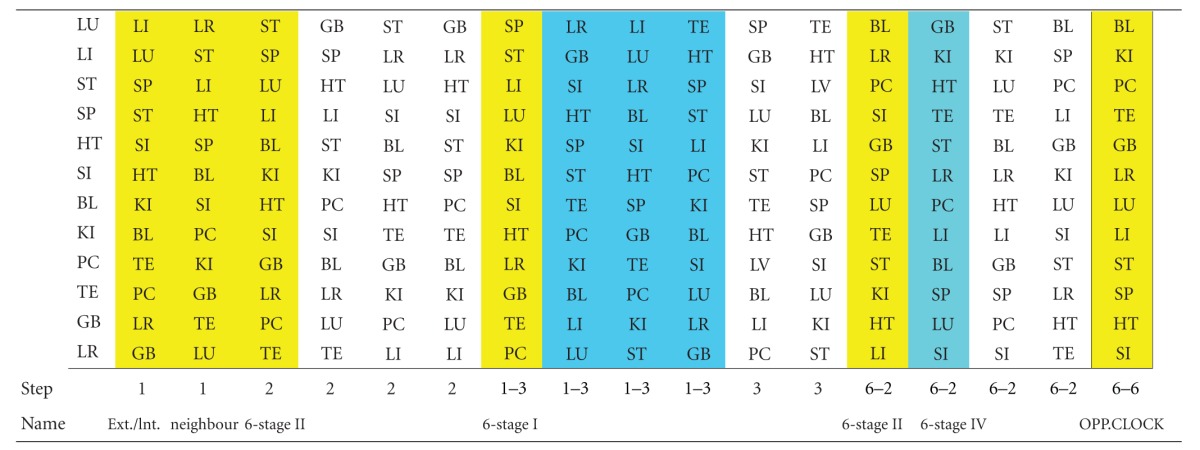

Yellow: historical systems, blue: new systems with additional combinations, and white: new systems without new combinations.

**Table 5:** Possible patterns for an ideal balanced 4-meridian combination.

−1, *x*, +1, +2
−2, −1, *x*, +1
*x*, +1, +2, +3
*x*, −1, −2, −3
*x*, +3, 6, −3

*x*: the balanced meridian, plus: step in the CC clockwise, minus: step in the CC counterclockwise.

**Table 6:** Research protocol for immediate pain relief in localised pain.

(1) Identify the affected meridian in the region of pain and ask the patient to characterize the pain on a visual analog scale (VAS) from 1 to 10.	
(2) Decide which unaffected extremity you want to treat first.	
(3) (a) *In the case that the chosen unaffected extremity is the contralateral arm of the painful arm or the contralateral leg of the painful leg, if the pain is on a yang meridian, the treatment has to applied to a yin meridian (or the other way around)*. (b) If the pain is on the upper extremities, but the chosen unaffected extremity is a lower extremity (or the other way around), you can decide whether you treat yin or yang meridians first.	
(4) After the decision on which extremity should be treated and whether yin or yang meridians have to be used, look up in Table 4 which meridians are the balancing meridians to the affected meridian.	
(5) (a) Palpate the balancing meridians in a corresponding body region by image or mirror (according to Figure 4) for Ashi points, decide which balancing meridian (according to Table 4) has the most painful Ashi points. Repeat this with other corresponding body regions (according to Figure 4) and decide which is the most painful area. (b) If you have followed variant (3b) you have to repeat the examination on the contralateral side of arm or leg. Treatment has to be applied to the more painful side.	
(6) Treat the most painful balancing meridian in the most painful corresponding area. Treat this Ashi area. Insert a needle into the most painful Ashi point. Apply further needles in acupuncture or Ashi points, usually 1-2 proximal the first needle and 1-2 distal the first point on the acupuncture meridian. The number of needles is dependent on the extension of the painful area on the affected meridian.	
(7) Move the affected joint or palpate the original region for pain and ask the patient about a change of symptoms using VAS. In case of 100% pain reduction, don't apply further needles.	
(8) Palpate another non affected extremity on balancing meridians (according to Table 4) in a corresponding body region by image or mirror (according to Figure 4) for Ashi points. But you have to stay in a balance of yin and yang between the legs or the arms. Repeat this with other corresponding body regions and decide on the most painful area.	
(9) See 6.	
(10) See 7.	
(11) Palpate the remaining non affected extremity on balancing meridians (according to Table 4) while staying in the yin-yang-balance of the extremities in a corresponding body region by image or mirroring (according to Figure 4) for Ashi points. Repeat this with other corresponding body regions and decide on the most painful area.	
(12) See 6.	
(13) See 7.	
(14) If the pain relief is still not sufficient, treatment can also applied to the affected meridian itself. But local treatment of the affected area can often produce discomfort to the patient, so the best choices are points by imaging or mirroring of corresponding areas (according to Figure 4) along the affected meridian itself. Repeat this with other corresponding body regions and decide on the most painful area.	
(15) See 6.	
(16) See 7.	
